# Tetra­aqua­bis­(piperazin-1-ium)cobalt(II) bis­(sulfate) dihydrate

**DOI:** 10.1107/S1600536813030365

**Published:** 2013-11-09

**Authors:** Thameur Sahbani, Wajda Smirani Sta, Mohamed Rzaigui

**Affiliations:** a Laboratoire de Chimie des Matériaux, Faculté des Sciences de Bizerte, 7021 Zarzouna Bizerte, Tunisia

## Abstract

In the centrosymmetric title compound, [Co(C_4_H_11_N_2_)_2_(H_2_O)_4_](SO_4_)_2_·2H_2_O, the Co^II^ atom is coordinated in a distorted octa­hedral geometry by four water O atoms and two piperazinium N atoms. These four water O atoms define an equatorial plane with a maximum deviation of 0.0384 (1) Å while the two piperazinium N atoms complete the octa­hedron in the axial positions. Neighboring complex mol­ecules and sulfate anions are connected through an extensive network of N—H⋯O and O—H⋯O hydrogen bonds, which link the different chemical species into layers in the *ab* plane. Additional O_water_—H⋯O hydrogen bonds involving the non-coordinating water mol­ecules and C—H⋯O inter­actions connect these layers into a three-dimensional supra­molecular structure.

## Related literature

For metal–sulfate complexes with piperazinium cations, see: Rekik *et al.* (2005 ▶); Pan *et al.* (2003 ▶); Sahbani *et al.* (2011 ▶); Mrinal *et al.* (2010 ▶). For the biological activity of piperazines, see: Bogatcheva *et al.* (2006 ▶). For a description of the Cambridge Structural Database, see: Allen (2002 ▶).
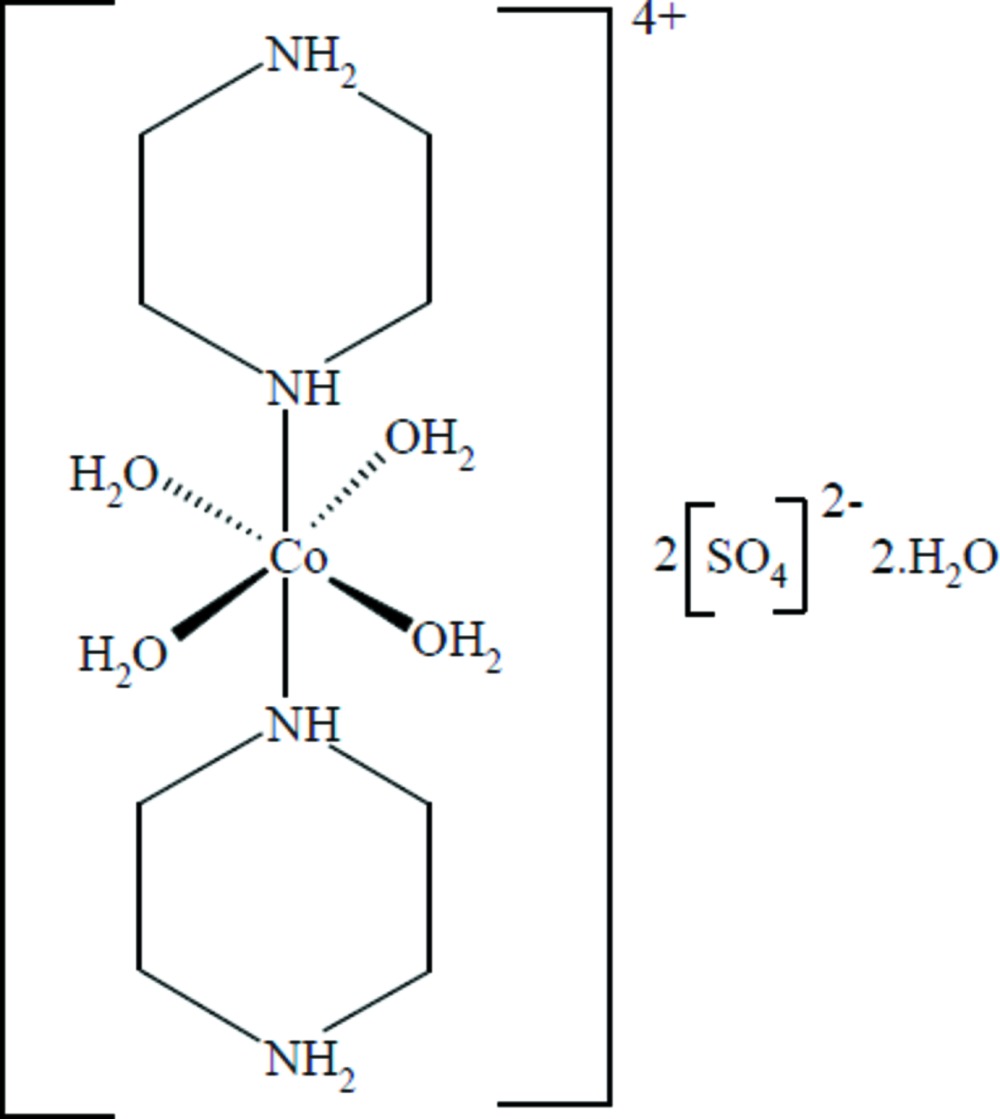


## Experimental

### 

#### Crystal data

[Co(C_4_H_11_N_2_)_2_(H_2_O)_4_](SO_4_)_2_·2H_2_O
*M* *_r_* = 533.46
Orthorhombic, 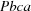

*a* = 12.187 (2) Å
*b* = 12.980 (2) Å
*c* = 13.437 (2) Å
*V* = 2125.5 (6) Å^3^
*Z* = 4
Ag *K*α radiation
λ = 0.56085 Å
μ = 0.56 mm^−1^
*T* = 293 K
0.30 × 0.20 × 0.20 mm

#### Data collection

Enraf–Nonius TurboCAD-4 diffractometer
8070 measured reflections
5211 independent reflections
3131 reflections with *I* > 2σ(*I*)
*R*_int_ = 0.023
2 standard reflections every 120 min intensity decay: 5%

#### Refinement

*R*[*F* ^2^ > 2σ(*F* ^2^)] = 0.045
*wR*(*F* ^2^) = 0.108
*S* = 1.04
5211 reflections
157 parameters
9 restraints
H-atom parameters constrained
Δρ_max_ = 0.73 e Å^−3^
Δρ_min_ = −0.35 e Å^−3^

### 

Data collection: *CAD-4 EXPRESS* (Enraf–Nonius, 1994 ▶); cell refinement: *CAD-4 EXPRESS*; data reduction: *XCAD4* (Harms & Wocadlo, 1995 ▶); program(s) used to solve structure: *SHELXS97* (Sheldrick, 2008 ▶); program(s) used to refine structure: *SHELXL97* (Sheldrick, 2008 ▶); molecular graphics: *ORTEP-3 for Windows* (Farrugia, 2012 ▶); software used to prepare material for publication: *WinGX* (Farrugia, 2012 ▶).

## Supplementary Material

Crystal structure: contains datablock(s) I, cad4. DOI: 10.1107/S1600536813030365/zl2569sup1.cif

Structure factors: contains datablock(s) I. DOI: 10.1107/S1600536813030365/zl2569Isup2.hkl

Additional supplementary materials:  crystallographic information; 3D view; checkCIF report

## Figures and Tables

**Table 1:** Hydrogen-bond geometry (Å, °)

*D*—H⋯*A*	*D*—H	H⋯*A*	*D*⋯*A*	*D*—H⋯*A*
O5—H1*O*5⋯O4^i^	0.83 (2)	1.96 (2)	2.776 (2)	170 (2)
O5—H2*O*5⋯O7	0.85 (2)	1.83 (2)	2.672 (2)	178 (2)
O7—H1*O*7⋯O3^ii^	0.82 (2)	1.98 (2)	2.797 (2)	169 (2)
O7—H2*O*7⋯O2	0.82 (2)	1.95 (2)	2.746 (2)	163 (2)
N1—H5⋯O3^iii^	0.91	2.35	3.240 (2)	166
O6—H1*O*6⋯O2^iv^	0.85 (2)	1.85 (2)	2.697 (2)	176 (3)
O6—H2*O*6⋯O1	0.84 (2)	1.86 (2)	2.686 (2)	168 (2)
N2—H9*A*⋯O1^v^	0.90	1.86	2.747 (2)	170
N2—H9*B*⋯O4^ii^	0.90	1.85	2.741 (2)	170
C3—H3*A*⋯O7^vi^	0.97	2.59	3.326 (3)	133
C4—H4*A*⋯O4^iv^	0.97	2.60	3.545 (2)	165

Symmetry codes: (i) 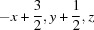; (ii) 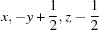; (iii) 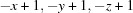; (iv) 
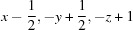
; (v) 
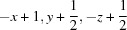
; (vi) 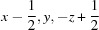.

**Fig. 1.**
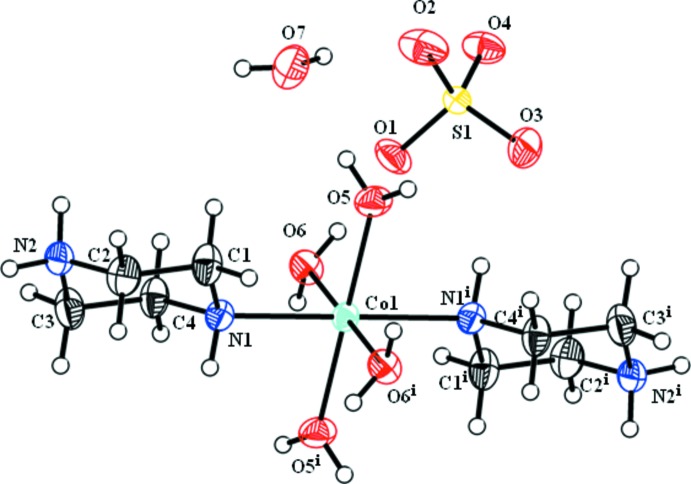
ORTEP-3 (Farrugia, 2012) view of [Co(C4H11N2)2(H2O)4](SO4)2.2H2O with atom numbering scheme. Displacement ellipsoids for non-H atoms are drawn at the 30% probability level. Symmetry code: (i) -x+1, -y+1, -z+1.

**Fig. 2.**
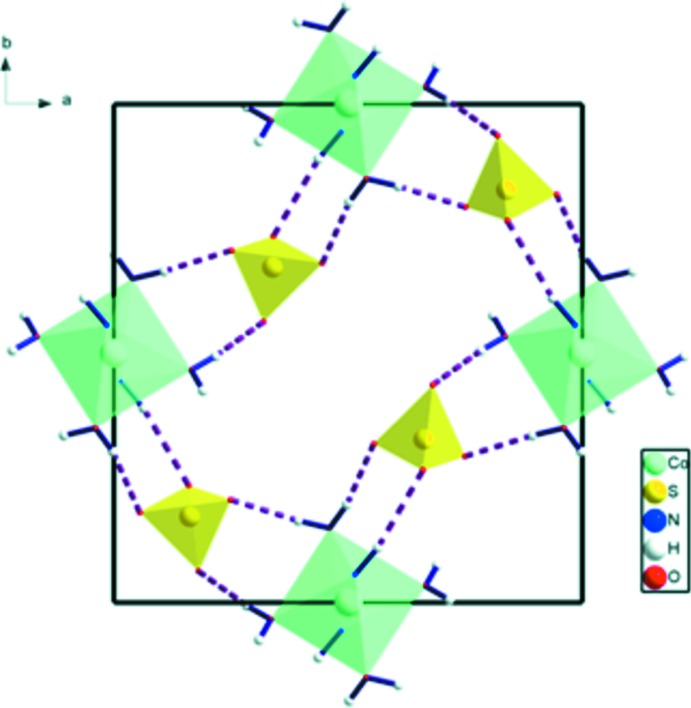
View of a corrugated layer of the title compound along the c axis. Sulfate polyhedra are shown in yellow and cobalt polyhedra in cyan. Hydrogen bonds are denoted by dashed lines.

**Fig. 3.**
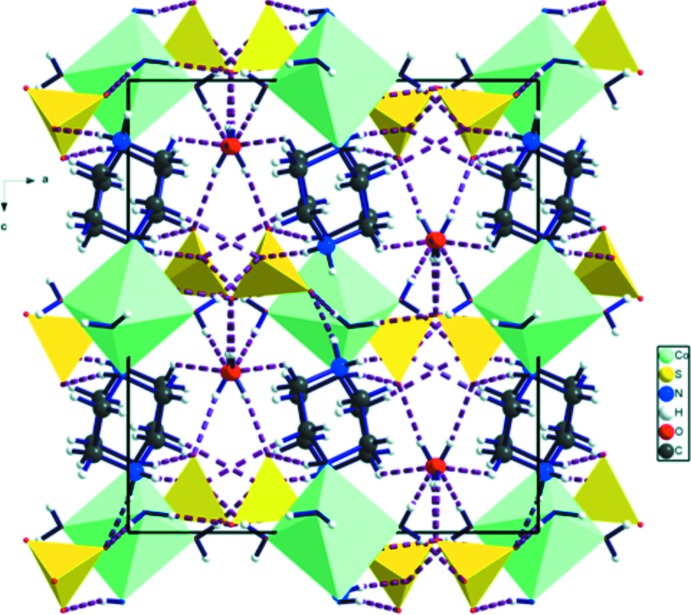
View of the atomic arrangement of the title compound along the b axis.

## supplementary crystallographic information

### 1. Comment

In view of the importance of piperazines which are found in biologically active materials across a number of areas of therapeutic importance (Bogatcheva *et al.*, 2006), the structural chemistry of metal salts that include piperazine and its derivatives does not cease to develop and continues to be a subject of research in many laboratories. Among the compounds investigated recently are several metal sulfate salts such as piperazinium hexaaquacobalt (II) disulfate (Pan *et al.*, 2003), piperazinedium hexaaquazinc (II) bis sulfate (Rekik *et al.*, 2005) and homopiperazin-1,4-diium bis-hexaaquacobalt (II) trisulfate (Sahbani *et al.*, 2011), among others. In these structures the cobalt atoms are hexacoordinated by six water molecules, and the piperazinium cations are diprotonated and not metal coordinated. In this report, we would like to present the crystal structure of a sulfate salt of a cobalt-piperazinium complex, [Co(C_4_H_11_N_2_)_2_(H_2_O)_4_](SO_4_)_2_.2H_2_O, in which the metal ion is coordinated to the piperazinium moiety, a coordination mode not reported so far for simple metal sulfates with piperazine as the only other ligand other than water.

The asymmetric unit of the title compound (I) consists of one half cationic complex [Co(C_4_H_11_N_2_)_2_(H_2_O)_4_]^4+^, an uncoordinated [SO_4_]^2-^ anion and one uncoordinated water molecule as shown in (Fig.1). The cobalt(II) ion, located on an inversion centre, exhibits a distorted octahedral coordination geometry with Co–O distances ranging from 2.0645 (14) to 2.0887 (13) Å and a Co–N bond distance equal to 2.2003 (14) Å. The O–Co–O and O–Co–N bond angles span the range from 85.72 to 94.28°. The piperazine nitrogen pairs from the monoprotonated cations are coordinated trans to each other, similar as described by Mrinal *et al.* (2010) for trans-[Ni(NCS)_4_(PpzH)_2_], and four oxygen atoms from the water molecules complete the coordination sphere of cobalt atom in the title compound. The piperazinium cation (C_4_H_11_N_2_)^+^ adopts a chair conformation as evidenced by the mean deviation (±0.0384 Å) from the least square plane defined by the four constituent atoms C1, C2, C3 and C4 and the remaining atoms N1 and N2 displaced from the plane by -0.6523 and 0.6392 Å, respectively. This is the so far only observed conformation for coordinated monoprotonated piperazines (19 entries in the Cambridge structural database with atom coordinates reported, database accessed Nov 2013 (Allen, 2002)).

Neighboring complexes and sulfate anions are connected through an extensive network of N—H···O and O—H···O hydrogen bonds, which link the different chemical species into two-dimensional layers in the ab plane (Fig.2). Additional OW—H···O hydrogen bonds that involve the not coordinated water molecules and C—H···O interactions connect these layers into a three-dimensional supramolecular structure.

### 2. Experimental

Piperazine (0.17g, 2 mmol) and cobalt acetate (0.24g, 1 mmol) were dissolved in 10 ml of water. The resulting solution was added to an aqueous solution of sulfuric acid (2 mmol, 2 ml). The mixture was stirred for 20 min at room temperature. After slow evaporation of the solvent over several days at ambient temperature, pink single crystals of the title compound suitable for X-ray diffraction formed in the solution. The crystals were filtered off, washed with a small amount of water and dried for 2 h. Yield: (60%, 21.32 mg). M.p. 270°C. Main IR bands (KBr disc, cm ^-1^): [vs = very strong; s = strong; w = weak] 3100 s, 3033 s, 2758 w, 1630 vs, 1467 s, 1342 s, 1225 w, 1040 s, 1080 vs, 963vs, 628 s, 490 s.

### 3. Refinement

All H atoms bonded to C and N atoms were positioned geometrically and treated as riding on their parent atoms, [N–H = 0.89, C–H =0.97 Å with *U*_iso_(H) = 1.2 *U*_eq_(C,N), but those attached to oxygen atom were located in difference density fourier maps. O–H bond distances and distances between two H atoms from each water molecule were restrained to be 0.85 (2) and 1.37 (2) Å, with *U*_iso_(H) = 1.5 *U*_eq_(O).

### Crystal data

**Table:**

[Co(C_4_H_11_N_2_)_2_(H_2_O)_4_](SO_4_)_2_·2H_2_O	*F*(000) = 1124
*M**_r_* = 533.46	*D*_x_ = 1.667 Mg m^−^^3^
Orthorhombic, *P**b**c**a*	Ag *K*α radiation, λ = 0.56085 Å
Hall symbol: -P 2ac 2ab	Cell parameters from 25 reflections
*a* = 12.187 (2) Å	θ = 9–11°
*b* = 12.980 (2) Å	µ = 0.56 mm^−^^1^
*c* = 13.437 (2) Å	*T* = 293 K
*V* = 2125.5 (6) Å^3^	Plate, pink
*Z* = 4	0.30 × 0.20 × 0.20 mm

### Data collection

**Table:**

Enraf–Nonius TurboCAD-4 diffractometer	*R*_int_ = 0.023
Radiation source: fine-focus sealed tube	θ_max_ = 28.0°, θ_min_ = 2.2°
Graphite monochromator	*h* = −2→20
non–profiled ω scans	*k* = −2→21
8070 measured reflections	*l* = −3→22
5211 independent reflections	2 standard reflections every 120 min
3131 reflections with *I* > 2σ(*I*)	intensity decay: 5%

### Refinement

**Table:**

Refinement on *F*^2^	Primary atom site location: structure-invariant direct methods
Least-squares matrix: full	Secondary atom site location: difference Fourier map
*R*[*F*^2^ > 2σ(*F*^2^)] = 0.045	Hydrogen site location: inferred from neighbouring sites
*wR*(*F*^2^) = 0.108	H-atom parameters constrained
*S* = 1.04	*w* = 1/[σ^2^(*F*_o_^2^) + (0.0459*P*)^2^ + 0.2397*P*] where *P* = (*F*_o_^2^ + 2*F*_c_^2^)/3
5211 reflections	(Δ/σ)_max_ = 0.001
157 parameters	Δρ_max_ = 0.73 e Å^−^^3^
9 restraints	Δρ_min_ = −0.35 e Å^−^^3^

### Special details

**Table:**

Geometry. All esds (except the esd in the dihedral angle between two l.s. planes) are estimated using the full covariance matrix. The cell esds are taken into account individually in the estimation of esds in distances, angles and torsion angles; correlations between esds in cell parameters are only used when they are defined by crystal symmetry. An approximate (isotropic) treatment of cell esds is used for estimating esds involving l.s. planes.
Refinement. Refinement of F^2^ against ALL reflections. The weighted R-factor wR and goodness of fit S are based on F^2^, conventional R-factors R are based on F, with F set to zero for negative F^2^. The threshold expression of F^2^ > 2sigma(F^2^) is used only for calculating R-factors(gt) etc. and is not relevant to the choice of reflections for refinement. R-factors based on F^2^ are statistically about twice as large as those based on F, and R- factors based on ALL data will be even larger.

### Fractional atomic coordinates and isotropic or equivalent isotropic displacement parameters (Å^2^)

**Table:**

	*x*	*y*	*z*	*U*_iso_*/*U*_eq_	
Co1	0.5000	0.5000	0.5000	0.02113 (7)	
S	0.66158 (3)	0.17244 (3)	0.58339 (3)	0.02297 (8)	
N1	0.48488 (11)	0.55679 (11)	0.34632 (10)	0.0247 (3)	
H5	0.4367	0.6102	0.3498	0.030*	
O5	0.66503 (11)	0.46657 (12)	0.48732 (11)	0.0344 (3)	
N2	0.51689 (12)	0.57886 (12)	0.13384 (11)	0.0290 (3)	
H9A	0.5015	0.6115	0.0764	0.035*	
H9B	0.5654	0.5285	0.1204	0.035*	
O1	0.55478 (12)	0.18124 (11)	0.53281 (12)	0.0414 (4)	
O4	0.68095 (11)	0.06305 (10)	0.60768 (11)	0.0363 (3)	
O3	0.65964 (14)	0.23468 (12)	0.67276 (11)	0.0488 (4)	
C4	0.43626 (16)	0.48409 (15)	0.27435 (13)	0.0312 (4)	
H4A	0.3677	0.4581	0.3010	0.037*	
H4B	0.4854	0.4260	0.2658	0.037*	
O2	0.74959 (13)	0.20647 (14)	0.51721 (13)	0.0508 (4)	
C1	0.58559 (16)	0.60093 (16)	0.30341 (13)	0.0332 (4)	
H1A	0.6392	0.5464	0.2948	0.040*	
H1B	0.6159	0.6505	0.3499	0.040*	
C2	0.56706 (18)	0.65320 (15)	0.20475 (14)	0.0346 (4)	
H2A	0.5188	0.7120	0.2135	0.041*	
H2B	0.6364	0.6778	0.1784	0.041*	
C3	0.41497 (16)	0.53265 (19)	0.17427 (14)	0.0375 (4)	
H3A	0.3878	0.4808	0.1285	0.045*	
H3B	0.3592	0.5855	0.1809	0.045*	
O6	0.46037 (12)	0.35031 (10)	0.45571 (11)	0.0303 (3)	
O7	0.74564 (16)	0.33382 (12)	0.35404 (12)	0.0458 (4)	
H1O5	0.7166 (17)	0.4944 (17)	0.5175 (17)	0.046 (8)*	
H2O5	0.6900 (18)	0.4253 (16)	0.4442 (14)	0.045 (7)*	
H1O7	0.714 (2)	0.320 (2)	0.3015 (14)	0.065 (9)*	
H2O7	0.745 (2)	0.2865 (17)	0.3943 (15)	0.061 (9)*	
H1O6	0.3931 (12)	0.3354 (18)	0.4629 (19)	0.049 (7)*	
H2O6	0.4975 (17)	0.3032 (18)	0.4830 (19)	0.063 (10)*	

### Atomic displacement parameters (Å^2^)

**Table:**

	*U*^11^	*U*^22^	*U*^33^	*U*^12^	*U*^13^	*U*^23^
Co1	0.02119 (12)	0.02247 (13)	0.01973 (12)	−0.00208 (12)	−0.00085 (12)	−0.00212 (12)
S	0.02149 (16)	0.02527 (17)	0.02214 (16)	0.00342 (15)	−0.00176 (15)	−0.00082 (16)
N1	0.0259 (7)	0.0270 (6)	0.0212 (6)	−0.0013 (5)	0.0007 (5)	−0.0003 (5)
O5	0.0243 (6)	0.0392 (7)	0.0397 (7)	0.0009 (6)	−0.0004 (6)	−0.0134 (6)
N2	0.0308 (8)	0.0341 (7)	0.0220 (6)	0.0027 (6)	0.0007 (6)	0.0045 (6)
O1	0.0327 (7)	0.0441 (8)	0.0474 (8)	0.0111 (7)	−0.0203 (6)	−0.0123 (7)
O4	0.0314 (7)	0.0282 (6)	0.0492 (8)	0.0052 (5)	0.0058 (6)	0.0087 (6)
O3	0.0609 (10)	0.0504 (9)	0.0351 (7)	0.0137 (8)	−0.0132 (7)	−0.0185 (7)
C4	0.0329 (8)	0.0378 (10)	0.0228 (7)	−0.0126 (8)	−0.0027 (7)	0.0021 (7)
O2	0.0375 (8)	0.0530 (9)	0.0618 (10)	0.0103 (7)	0.0192 (8)	0.0279 (8)
C1	0.0326 (9)	0.0417 (10)	0.0251 (8)	−0.0134 (8)	−0.0025 (7)	0.0028 (8)
C2	0.0422 (10)	0.0318 (9)	0.0297 (8)	−0.0085 (8)	0.0021 (8)	0.0068 (8)
C3	0.0317 (9)	0.0535 (11)	0.0271 (8)	−0.0073 (9)	−0.0071 (8)	0.0049 (9)
O6	0.0314 (6)	0.0253 (6)	0.0342 (7)	−0.0010 (5)	−0.0061 (6)	−0.0021 (5)
O7	0.0644 (10)	0.0405 (8)	0.0324 (7)	0.0014 (8)	0.0014 (8)	−0.0016 (7)

### Geometric parameters (Å, º)

**Table:**

Co1—O5	2.0645 (14)	N2—H9A	0.9000
Co1—O5^i^	2.0645 (14)	N2—H9B	0.9000
Co1—O6	2.0887 (13)	C4—C3	1.508 (2)
Co1—O6^i^	2.0887 (13)	C4—H4A	0.9700
Co1—N1^i^	2.2003 (14)	C4—H4B	0.9700
Co1—N1	2.2003 (14)	C1—C2	1.506 (3)
S—O3	1.4475 (15)	C1—H1A	0.9700
S—O2	1.4615 (15)	C1—H1B	0.9700
S—O1	1.4728 (14)	C2—H2A	0.9700
S—O4	1.4759 (13)	C2—H2B	0.9700
N1—C1	1.472 (2)	C3—H3A	0.9700
N1—C4	1.475 (2)	C3—H3B	0.9700
N1—H5	0.9100	O6—H1O6	0.848 (15)
O5—H1O5	0.831 (15)	O6—H2O6	0.845 (16)
O5—H2O5	0.846 (15)	O7—H1O7	0.824 (16)
N2—C3	1.483 (2)	O7—H2O7	0.819 (15)
N2—C2	1.488 (2)		
			
O5—Co1—O5^i^	180.0	C2—N2—H9A	109.2
O5—Co1—O6	90.35 (6)	C3—N2—H9B	109.2
O5^i^—Co1—O6	89.65 (6)	C2—N2—H9B	109.2
O5—Co1—O6^i^	89.65 (6)	H9A—N2—H9B	107.9
O5^i^—Co1—O6^i^	90.35 (6)	N1—C4—C3	112.73 (15)
O6—Co1—O6^i^	180.0	N1—C4—H4A	109.0
O5—Co1—N1^i^	85.72 (6)	C3—C4—H4A	109.0
O5^i^—Co1—N1^i^	94.28 (6)	N1—C4—H4B	109.0
O6—Co1—N1^i^	88.57 (5)	C3—C4—H4B	109.0
O6^i^—Co1—N1^i^	91.43 (5)	H4A—C4—H4B	107.8
O5—Co1—N1	94.28 (6)	N1—C1—C2	113.27 (16)
O5^i^—Co1—N1	85.72 (6)	N1—C1—H1A	108.9
O6—Co1—N1	91.43 (5)	C2—C1—H1A	108.9
O6^i^—Co1—N1	88.57 (5)	N1—C1—H1B	108.9
N1^i^—Co1—N1	180.0	C2—C1—H1B	108.9
O3—S—O2	110.37 (11)	H1A—C1—H1B	107.7
O3—S—O1	108.98 (9)	N2—C2—C1	109.46 (15)
O2—S—O1	110.14 (10)	N2—C2—H2A	109.8
O3—S—O4	110.86 (9)	C1—C2—H2A	109.8
O2—S—O4	107.94 (9)	N2—C2—H2B	109.8
O1—S—O4	108.54 (8)	C1—C2—H2B	109.8
C1—N1—C4	109.08 (14)	H2A—C2—H2B	108.2
C1—N1—Co1	115.35 (11)	N2—C3—C4	110.60 (15)
C4—N1—Co1	115.76 (10)	N2—C3—H3A	109.5
C1—N1—H5	105.2	C4—C3—H3A	109.5
C4—N1—H5	105.2	N2—C3—H3B	109.5
Co1—N1—H5	105.2	C4—C3—H3B	109.5
Co1—O5—H1O5	127.2 (16)	H3A—C3—H3B	108.1
Co1—O5—H2O5	122.7 (16)	Co1—O6—H1O6	113.8 (17)
H1O5—O5—H2O5	109.7 (19)	Co1—O6—H2O6	115.2 (19)
C3—N2—C2	111.85 (15)	H1O6—O6—H2O6	107.6 (19)
C3—N2—H9A	109.2	H1O7—O7—H2O7	114 (2)

Symmetry code: (i) −*x*+1, −*y*+1, −*z*+1.

### Hydrogen-bond geometry (Å, º)

**Table:**

*D*—H···*A*	*D*—H	H···*A*	*D*···*A*	*D*—H···*A*
O5—H1*O*5···O4^ii^	0.83 (2)	1.96 (2)	2.776 (2)	170 (2)
O5—H2*O*5···O7	0.85 (2)	1.83 (2)	2.672 (2)	178 (2)
O7—H1*O*7···O3^iii^	0.82 (2)	1.98 (2)	2.797 (2)	169 (2)
O7—H2*O*7···O2	0.82 (2)	1.95 (2)	2.746 (2)	163 (2)
N1—H5···O3^i^	0.91	2.35	3.240 (2)	166
O6—H1*O*6···O2^iv^	0.85 (2)	1.85 (2)	2.697 (2)	176 (3)
O6—H2*O*6···O1	0.84 (2)	1.86 (2)	2.686 (2)	168 (2)
N2—H9*A*···O1^v^	0.90	1.86	2.747 (2)	170
N2—H9*B*···O4^iii^	0.90	1.85	2.741 (2)	170
C3—H3*A*···O7^vi^	0.97	2.59	3.326 (3)	133
C4—H4*A*···O4^iv^	0.97	2.60	3.545 (2)	165

Symmetry codes: (i) −*x*+1, −*y*+1, −*z*+1; (ii) −*x*+3/2, *y*+1/2, *z*; (iii) *x*, −*y*+1/2, *z*−1/2; (iv) *x*−1/2, −*y*+1/2, −*z*+1; (v) −*x*+1, *y*+1/2, −*z*+1/2; (vi) *x*−1/2, *y*, −*z*+1/2.

## References

[bb1] Allen, F. H. (2002). *Acta Cryst.* B**58**, 380–388. 10.1107/s0108768102003890 12037359

[bb2] Bogatcheva, E., Hanrahan, C., Nikonenko, B., Samala, R., Chen, P., Gearhart, J., Barbosa, F., Einck, L., Nacy, C. A. & Protopopova, M. (2006). *J. Med. Chem.* **49**, 3045–3048. 10.1021/jm050948+ PMC4869334 16722620

[bb3] Enraf–Nonius (1994). *CAD-4 EXPRESS* Enraf–Nonius, Delft, The Netherlands.

[bb4] Farrugia, L. J. (2012). *J. Appl. Cryst.* **45**, 849–854.

[bb5] Harms, K. & Wocadlo, S. (1995). *XCAD4* University of Marburg, Germany.

[bb6] Mrinal, S., Supriti, P., Kanak, R., Sandipan, R., Avijit, S., Alok, K. & Debashis, R. (2010). *Inorg. Chim. Acta*, **363**, 3041–3047.

[bb7] Pan, J.-X., Yang, G.-Y. & Sun, Y.-Q. (2003). *Acta Cryst.* E**59**, m286–m288.

[bb8] Rekik, W., Naïli, H., Mhiri, T. & Bataille, T. (2005). *Acta Cryst.* E**61**, m629–m631.

[bb9] Sahbani, T., Smirani Sta, W., S. Al-Deyab, S. & Rzaigui, M. (2011). *Acta Cryst.* E**67**, m1079. 10.1107/S1600536811027012 PMC3212155 22090857

[bb10] Sheldrick, G. M. (2008). *Acta Cryst.* A**64**, 112–122. 10.1107/S0108767307043930 18156677

